# Predicting academic outcomes in an Australian graduate entry medical programme

**DOI:** 10.1186/1472-6920-14-31

**Published:** 2014-02-15

**Authors:** Ian B Puddey, Annette Mercer

**Affiliations:** 1 Faculty of Medicine, Dentistry and Health Sciences, University of Western Australia, 35 Stirling Hwy, Crawley, WA 6009, Australia

## Abstract

**Background:**

Predictive validity studies for selection criteria into graduate entry courses in Australia have been inconsistent in their outcomes. One of the reasons for this inconsistency may have been failure to have adequately considered background disciplines of the graduates as well as other potential confounding socio-demographic variables that may influence academic performance.

**Methods:**

Graduate entrants into the MBBS at The University of Western Australia between 2005 and 2012 were studied (N = 421). They undertook a 6-month bridging course, before joining the undergraduate-entry students for Years 3 through 6 of the medical course. Students were selected using their undergraduate Grade Point Average (GPA), Graduate Australian Medical School Admissions Test scores (GAMSAT) and a score from a standardised interview. Students could apply from any background discipline and could also be selected through an alternative rural entry pathway again utilising these 3 entry scores. Entry scores, together with age, gender, discipline background, rural entry status and a socioeconomic indicator were entered into linear regression models to determine the relative influence of each predictor on subsequent academic performance in the course.

**Results:**

Background discipline, age, gender and selection through the rural pathway were variously related to each of the 3 entry criteria. Their subsequent inclusion in linear regression models identified GPA at entry, being from a health/allied health background and total GAMSAT score as consistent independent predictors of stronger academic performance as measured by the weighted average mark for the core units completed throughout the course. The Interview score only weakly predicted performance later in the course and mainly in clinically-based units. The association of total GAMSAT score with academic performance was predominantly dictated by the score in GAMSAT Section 3 (Reasoning in the biological and physical sciences) with Section 1 (Reasoning in the humanities and social sciences) and Section 2 (Written communication) also contributing either later or early in the course respectively. Being from a more disadvantaged socioeconomic background predicted weaker academic performance early in the course. Being an older student at entry or from a humanities background also predicted weaker academic performance.

**Conclusions:**

This study confirms that both GPA at entry and the GAMSAT score together predict outcomes not only in the early stages of a graduate-entry medical programme but throughout the course. It also indicates that a comprehensive evaluation of the predictive validity of GAMSAT scores, interview scores and undergraduate academic performance as valid selection processes for graduate entry into medical school needs to simultaneously consider the potential confounding influence of graduate discipline background and other socio-demographic factors on both the initial selection parameters themselves as well as subsequent academic performance.

## Background

Graduate entry medical programmes in Australia have increased in number from three in the early 1990s to twelve currently. Elliot and Epstein [1] in discussing the reasons behind this increase in graduate-entry programs, concluded that they provide an important alternative, but not a replacement, for undergraduate-entry medical schools. The graduate-entry programs usually consist of four years of study and are available to relatively high performing graduates who have achieved a specified Grade Point Average (GPA) during their undergraduate studies. At present all students applying to enter a graduate entry medical course in Australia must also sit the Graduate Australian Medical Schools Admissions Test (GAMSAT), a cognitive aptitude test consisting of three sections: Section 1 -*Reasoning in the humanities and social sciences,* Section 2 *- Written communication* and Section 3 - *Reasoning in the biological and physical sciences.* An overall GAMSAT score is calculated using the three section scores in the ratio 1:1:2. In addition, with the exception of one university, applicants are required to undertake an interview. Medical schools use some or all of three entry criteria (GPA, GAMSAT score and interview score) in a variety of ways to make their final selection of students. Some universities have an additional criterion, such as a personal statement.

The GAMSAT was developed by the Australian Council for Educational Research (ACER) under contract to the GAMSAT Consortium, to overcome the difficulties in distinguishing between the GPAs from a range of courses and a range of universities. It was first administered in 1995 and seeks to provide a ‘level playing field’ to select an intentionally heterogeneous cohort [1]. Section 3 (Reasoning in the biological and physical sciences), which is double weighted in calculating the final GAMSAT score, is useful in providing a guarantee of some competence in science across a variety of applicants’ academic backgrounds.

A number of studies have been published in recent years investigating the predictive validity of the entry criteria for graduate entry medical courses and in particular the GAMSAT. An initial study by Groves et al. [2] in medical students from 2 universities showed mixed, but weak, relationships between both GAMSAT and interview results and in-course tests of Clinical Reasoning and Diagnostic Thinking as well as Year 2 examination results causing them to challenge the predictive validity of both these selection factors. However, although finding weaker academic performance in those from non-biological vs biological discipline backgrounds they did not consider a potential confounding influence of the well established effect of academic background on performance in the GAMSAT [3]. A larger study by Coates [4] included students from 6 universities and concluded that GAMSAT and GPA scores, but not an interview score, each uniquely predicted first-year performance. A study at Queensland University [5] showed selection criteria to be only modest predictors of performance in the course with GPA the strongest followed by interview score and GAMSAT scores, the latter two being relatively weak in their associations with course performance. In a further recent study Bodger et al. [6] reported in a multivariate factor analysis that only past academic record and not a “GAMSAT” factor or an “Interview” factor was correlated with course performance. Their analysis was however, limited to a self-selected cohort of 105 students and only assessed performance in the first 2 years of the course. They found no relationship between performance in the course and prior academic background but did not consider an effect of prior academic background on performance in the GAMSAT as a possible confounding variable in their analysis.

Recent reports have also raised the possibility that there may be potential socio-economic influences on performance in aptitude tests such as the GAMSAT that have not been considered in previous predictive validity studies. Socio-economic factors were identified as determinants of performance in both the Medical College Admission Test (MCAT) [7], used for medical student selection in North America, and the UK Clinical Aptitude test (UKCAT), used in medical student selection in the UK [8,9]. In addition we have recently reported that better performance by candidates in the Undergraduate Medicine and Health Sciences Admission Test (UMAT), widely used for medical school admission in Australia and New Zealand, is clearly linked to an increase in socio-economic advantage and reduced disadvantage [10].

### Background to this study

At the University of Western Australia (UWA), the MBBS for school-leaver entry is currently a six-year course integrating biomedical science and clinical teaching. In 2004 the Faculty of Medicine, Dentistry and Health Sciences undertook its first round of selection of a graduate entry stream into the existing undergraduate medical course. The new programme, known as the Graduate Entry Medical Program (GEMP), consisted of a 6-month bridging course followed by Levels 3 to 6 of the undergraduate course. In the bridging course the biological, psychological, social, clinical and population aspects of health and illness are studied in preparation for Level 3, the final pre-clinical year before the more clinically-focussed Levels 4 – 6.

Selection into the GEMP at UWA comprises a two-stage process. Candidates are selected for interview using their GPA and overall GAMSAT score, combined with equal weight. A minimum GPA of 5.5 and a minimum score of 50 on the overall GAMSAT score and on each section are required. The UWA interview is a highly structured panel interview with a focus on communication skills and a comprehensive description of the interview process has been previously published [11]. Following interview the three criteria (GPA, GAMSAT score and interview score) are weighted equally to produce a final ranked list from which offers are made. Students can apply from any background discipline and can also be selected through an alternative rural entry pathway again utilising these 3 entry scores. In 2005 the first cohort of 19 GEMP students entered the bridging course, followed in 2006 by 37 students and subsequently by approximately 60 students each year. The aim of the study was to determine the relationship between the students’ entry scores, demographic characteristics and background discipline with subsequent performance in the course. This study is part of a larger project which includes the school-leaver entrants who undertake the full 6-year course. The results of that part of the project have been reported elsewhere [11]. The project has been approved by the Human Research Ethics Committee at UWA (file reference RA/4/1/2178).

## Methods

In this study all the students who entered the GEMP from 2005 to 2012 (N = 421) were included except for indigenous and international students who were admitted through alternative selection pathways. The first five cohorts had completed the course (N = 219), the 2012 cohort had completed the bridging course and Level 3 and all other cohorts were at varying stages throughout levels 3 – 6 of the 6-year MBBS. Those studied included any who subsequently withdrew (N = 11) or were excluded for unsatisfactory progress (N = 5). In cases where students had repeated a unit their first unit score was included in the analysis.

A database was established consisting of the following sets of variables: entry scores, demographics, discipline of first degree and outcome measures for performance in the bridging course and Levels 3–6 of the MBBS (Table 1). The Weighted Average Mark (WAM) calculated for each of these levels used results (expressed as percentages) for core units weighted by the UWA points system for the size of the unit. As well as WAM for each level an overall WAM was calculated from the accumulated results of all core units for those students who had completed the course. At the time of analysis 219 students had completed the course with the remainder still progressing through the course. Secondary outcome variables included the mark for a range of individual units selected to assess the relative contribution to the WAM of performance in specific units that were either knowledge-based (where the curriculum was delivered mainly in didactic fashion in lectures, tutorials and laboratory sessions; and assessment was predominantly of factual knowledge), or clinically based (with the curriculum delivered through a combination of problem-based learning tutorials, case-based tutorials or clinical teaching; and assessment was either through a multidisciplinary observed structured clinical examination or a composite assessment of clinical performance).

**Table 1:** Variables entered in regression models for the study

**Entry scores**	**Demographics**	**Outcome measures**
Grade Point Average (GPA) value on a continuous 7-point scale	Age in years	Weighted Average Mark (WAM) of all core units completed
GAMSAT overall score	Sex (0 = F, 1 = M)	WAM of all core units in the bridging course
GAMSAT Section 1	Background discipline:	WAM of all core units in Levels 3, 4, 5 and 6
Reasoning in the Humanities and social sciences	(0 = No, 1 = Yes)	*Weighting was according to the size of the units and all results were recorded as percentages.*
GAMSAT Section 2	• Health/Allied health	
Written communication	• Humanities/Law/Business/Commerce	
GAMSAT Section 3	• Physical sciences	
Reasoning in the biological and physical sciences	Index of relative socioeconomic advantage & disadvantage decile	
Interview score	(0 = 1st-8th Decile, 1 = 9th-10th Decile)	
	Rural entry (0 = No, 1 = Yes)	

The discipline/major of the first degree was classified into one of the four categories: Biological sciences/science, Health/allied health, Humanities/law/business/commerce and Physical sciences. Dummy variables for each discipline were constructed for subsequent entry into multivariate linear regression models. Mathematics and IT were classified as Physical sciences while Psychology was classified with the Humanities if students graduated with a Bachelor of Arts or with Biological Sciences/science if they graduated with either a Bachelor of Science or a double degree in both Arts and Science.

As a socioeconomic indicator, the correspondence postcode at entry for each student was linked to the Index of Relative Socioeconomic Advantage and Disadvantage (IRSAD) score from the Australian 2006 census Socio-Economic Indices for Areas (SEIFA) [12]. The IRSAD score is derived by principal components analysis of 21 separate variables such as low or high income, internet connection, unemployment, occupation and education. The score is standardised against a mean of 1000 with a standard deviation of 100 with two thirds of SEIFA scores falling between 900 and 1100.The IRSAD decile score is utilised in regression because SEIFA codes are not linear. A dummy variable was constructed which dichotomised the cohort into the top 2 deciles vs the bottom 8 deciles because two thirds of the study population were within the top 2 deciles with increasingly smaller numbers across the other 8 deciles.

### Statistics

Data were analysed using IBM SPSS Statistics Release 20.0.0. Pearson correlations (and point bi-serial correlations for the dichotomised variables) were calculated between the predictor variables (entry scores and demographics) and the outcome variables. Performance in the GAMSAT or during the course by disciple background was analysed by oneway ANOVA with post hoc comparisons using the Bonferroni correction. Backwards linear regression models were constructed for each outcome variable using the full set of predictor variables listed in Table 1. Models included either the total GAMSAT score or all three sections of GAMSAT entered separately. The estimates obtained from the linear regression models are reported without correction.

## Results

### Summary statistics

Summary statistics for predictor variables, demographic variables and outcome variables are shown in Table 2.

**Table 2:** Summary statistics for key variables

	**N**	**Mean ± SEM or % of total N**
**Demographic variables**		
Age (yr)	421	25.7 ± 0.3
Sex (Female/Male)	421	56.3%/43.7%
Rural entry (No/Yes)	421	81.5%/18.5%
Index of relative socioeconomic advantage & disadvantage decile (Bottom 8 deciles/Top 2 deciles)	417	36.8%/62.2%
Discipline background:		
Biological sciences	421	63.7%
Health/Allied health	421	21.1%
Humanities/Law/Business/Commerce	421	8.8%
Physical sciences	421	6.4%
**Entry scores**		
GPA	421	6.38 ± 0.02
Interview score	421	27.3 ± 0.3
Total GAMSAT score	421	61.8 ± 0.3
GAMSAT 1 score (Reasoning in the humanities and social sciences)	421	60.1 ± 0.3
GAMSAT 2 score (Written communication)	421	62.2 ± 0.4
GAMSAT 3 score (Reasoning in the biological and physical sciences)	421	62.2 ± 0.4
**Outcome variables**		
WAM core units levels 2-6	219	70.1 ± 0.3
WAM level 2 bridging course	421	72.4 ± 0.4
WAM level 3	414	72.8 ± 0.4
WAM level 4	344	72.1 ± 0.3
WAM level 5	276	73.0 ± 0.3
WAM level 6	219	73.0 ± 0.3

### Univariate analyses of entry scores

Correlations between the demographic predictor variables and entry scores are outlined in Table 3. Older subjects, those who entered via the rural pathway and those from a health/allied health discipline background had a lower GPA at entry while for those from a biological sciences/science background it was higher. The interview score was lower for those entering via the rural pathway. Both female students and rural entry students had a lower total GAMSAT score, mainly because of relatively weaker performance in Section 3 - Reasoning in the biological and physical sciences. There was no correlation between the Index of Relative Socioeconomic Advantage and Disadvantage Score and any of the selection criteria.

**Table 3:** Pearson correlations (and point bi-serial correlations for the dichotomised variables) between the predictor variables and entry scores (N = 421)

		***GPA at entry***	***Total GAMSAT score***	***GAMSAT 1: Reasoning in the humanities and social sciences***	***GAMSAT 2: Written communication***	***GAMSAT 3: Reasoning in the biological and physical sciences***	***Interview score***
***Age at entry***	Correlation	−0.136	−0.035	0.165	0.278	−0.216	0.118
	P value	**0.005**	0.468	**0.001**	**<0.001**	**<0.001**	**0.016**
***Sex (0 = F, 1 = M)***	Correlation	−0.090	0.209	0.079	0.064	0.213	−0.009
	P value	0.067	**<0.001**	0.106	0.191	**<0.001**	0.852
***Biological science/Science (No = 0, Yes = 1)***	Correlation	0.121	0.119	−0.052	−0.126	0.231	−0.033
	P value	**0.013**	**0.015**	0.285	**0.010**	**<0.001**	0.497
***Health/Allied health (No = 0, Yes = 1)***	Correlation	−0.108	−0.229	−0.132	−0.003	−0.251	0.040
	P value	**0.027**	**<0.001**	**0.012**	0.722	**<0.001**	0.358
***Humanities/Commerce/Business/ Law (No = 0, Yes = 1)***	Correlation	−0.087	0.006	0.211	0.238	−0.164	0.074
	P value	0.076	0.904	**<0.001**	**<0.001**	**0.001**	0.128
***Physical sciences (No = 0, Yes = 1)***	Correlation	0.043	0.143	0.078	−0.024	0.158	−0.089
	P value	0.374	**0.003**	0.110	0.620	**0.001**	0.069
***Rural entry (No = 0, Yes = 1)***	Correlation	−0.165	−0.120	0.002	0.007	−0.157	−0.124
	P value	**0.001**	**0.014**	0.965	0.885	**0.001**	**0.011**
***IRSAD decile (0 = 1st-8th decile, 1 = 9th–10th decile)***	Correlation	0.087	0.050	0.028	0.053	0.033	0.036
	P value	0.075	0.305	0.570	0.277	0.495	0.466

(Significant P-values are boldfaced).

**Figure 1:**
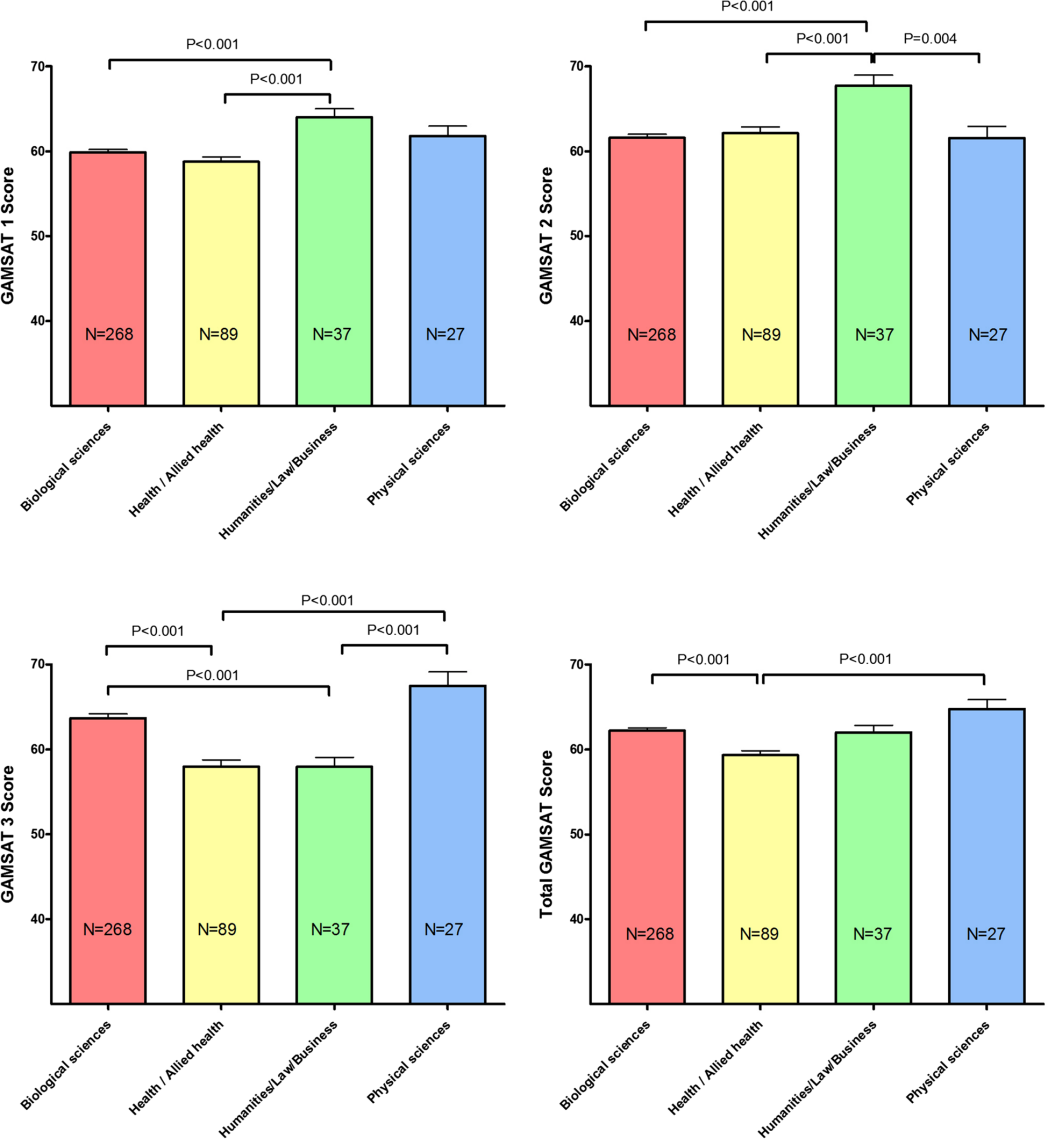
GAMSAT and GAMSAT component scores by background discipline (P-values are from Oneway ANOVA - post hoc comparisons with Bonferroni correction).

Relative performance in the GAMSAT and each of its components by discipline background is depicted in Figure 1. Students from a physical sciences background performed best in total GAMSAT predominantly because of a stronger performance in Section 3. In students from a humanities background a stronger performance in both Section 1 of the GAMSAT (Reasoning in the humanities and social sciences) and Section 2 (Written communication) was counterbalanced by a weaker performance in Section 3. In students from a biological sciences/science background a weaker performance in Section 2 was counterbalanced by a stronger performance in Section 3. Students from a health/allied health background achieved the lowest total GAMSAT scores at entry predominantly because of a weaker performance in both Section 1 and Section 3.

### Univariate analyses of outcome variables

Correlations between all predictor variables and the outcome variables are summarised in Table 4. GPA at entry was the strongest and most consistent correlate of academic performance, but waned in relative strength as the course progressed. There were no significant univariate associations of academic performance with entry interview scores or rural entry status. Age at entry was negatively correlated with academic performance for the overall WAM and in Levels 2 to 5 of the course. Gender was largely unrelated to academic performance except in Level 5 of the course where females performed better overall. Throughout the course the performance of students from a Humanities/law/business/commerce background was significantly weaker than those from all other backgrounds (Overall WAM 69.0 ± 0.8 vs 72.4 ± 0.3, P = 0.001). In the latter years of the course the performance of biological sciences/science students was also consistently weaker than that of health/allied health students. Approximately two-thirds of the students came from postcode areas within the top 2 deciles of higher socioeconomic advantage and lower socioeconomic disadvantage for the Australian population. When compared to those from the lower 8 deciles their academic performance was higher (Overall WAM 72.7 ± 0.5, N = 135 vs 71.1 ± 0.4, N = 84, P = 0.015). This was more evident in the earlier stages of the course but no longer significant in levels 4 and 6 of the course. Total GAMSAT score was correlated with academic performance from Levels 2 to 5 with this association again stronger in the earlier years. It was also correlated with the overall WAM for those who had completed the course. These correlations were largely driven by the associations with Section 3 of the GAMSAT, with Section 1 also contributing at Level 5.

**Table 4:** Pearson correlations (and point bi-serial correlations for the dichotomised variables) between the predictor variables and the outcome variables

		***WAM core units overall (N = 219)***	***WAM level 2(N = 421)***	***WAM level 3 (N = 414)***	***WAM level 4 (N = 344)***	***WAM level 5 (N = 276)***	***WAM level 6 (N = 219)***
***Age at entry***	Correlation	−0.167	−0.220	−0.162	−0.139	−0.275	−0.099
	P value	**0.013**	**<0.001**	**0.001**	**0.010**	**<0.001**	0.146
***Sex (0 = F, 1 = M)***	Correlation	−0.067	0.022	−0.064	−0.060	−0.158	0.033
	P value	0.326	0.649	0.192	0.270	**0.009**	0.623
***Biological science/Science background (No = 0, Yes = 1)***	Correlation	−0.009	0.057	−0.006	−0.097	−0.032	−0.109
	P value	0.892	0.242	0.906	0.074	0.601	0.107
***Health/Allied health background *** ***(No = 0, Yes = 1)***	Correlation	0.174	0.073	0.087	0.206	0.187	0.229
	P value	**0.010**	0.135	0.077	**<0.001**	**0.002**	**0.001**
***Humanities/Commerce/Business/ Law background (No = 0, Yes = 1)***	Correlation	−0.233	−0.212	−0.148	−0.137	−0.225	−0.174
	P value	**0.001**	**<0.001**	**0.002**	**0.011**	**<0.001**	**0.010**
***Physical sciences background*** *** (No = 0, Yes = 1)***	Correlation	0.014	0.013	0.036	−0.006	0.015	0.047
	P value	0.833	0.783	0.465	0.915	0.810	0.485
***GPA at entry***	Correlation	0.270	0.389	0.384	0.272	0.198	0.109
	P value	**<0.001**	**<0.001**	**<0.001**	**<0.001**	**0.001**	0.108
***Rural entry*** ***(No = 0, Yes = 1)***	Correlation	−0.049	−0.091	−0.095	0.030	−0.102	−0.046
	P value	0.467	0.061	0.053	0.575	0.090	0.501
***IRSAD_Decile_x (0 =1st-8th decile, 1 = 9th-10th decile)***	Correlation	0.164	0.151	0.181	0.102	0.138	0.028
	P value	**0.015**	**0.002**	**<0.001**	0.059	**0.022**	0.683
***Interview score***	Correlation	0.077	−0.041	−0.061	0.014	0.033	0.122
	P value	0.257	0.398	0.215	0.791	0.585	0.071
***Total GAMSAT score***	Correlation	0.268	0.282	0.216	0.117	0.189	0.065
	P value	**<0.001**	**<0.001**	**<0.001**	**0.030**	**0.002**	0.339
***GAMSAT 1 - Reasoning in the humanities and social sciences***	Correlation	0.156	0.069	0.042	0.067	0.162	0.046
	P value	**0.021**	0.158	0.391	0.214	**0.007**	0.496
***GAMSAT 2 - Written communication***	Correlation	0.058	0.056	0.071	0.024	−0.017	0.027
	P value	0.358	0.254	0.148	0.654	0.783	0.688
***GAMSAT 3 - Reasoning in the biological and physical sciences***	Correlation	0.271	0.311	0.229	0.117	0.194	0.061
	P value	**<0.001**	**<0.001**	**<0.001**	**0.030**	**0.001**	0.369

(WAM = Weighted average mark, IRSAD – index of relative socioeconomic advantage and disadvantage**,** Significant P-values are boldfaced).

### Multvariate analyses of outcome variables

The final backward multivariate linear regression models for the relationship between selection factors and demographic characteristics with academic performance throughout the course are outlined in Table 5. GPA at entry, being from a health/allied health background and total GAMSAT score were the most consistent independent predictors of stronger academic performance as measured by the weighted average mark for the core units completed throughout the course. Being from a humanities background predicted a lower overall WAM and was an independent predictor of a weaker performance at Level 2 and Level 5. Being from a more disadvantaged socioeconomic background predicted weaker academic performance predominantly in the early years of the course while being an older student at entry predicted weaker academic performance throughout the course. In contrast to the univariate analysis, multivariate analysis indicated that the interview score weakly predicted performance at level 5 and 6 of the course. This relationship was not seen in the knowledge-based units (Table 6) but predominantly in the clinically-based units (Table 7). In a similar fashion, females performed better than males for WAM at Level 5 of the course (Table 5) and in all clinically based units (Table 7). The association of total GAMSAT score with academic performance was predominantly dictated by the score in GAMSAT Section 3 (Reasoning in the biological and physical sciences) with Section 2 (Written communication) contributing in multivariate analysis early in the course and Section 1 (Reasoning in the humanities and social sciences) in the latter years (Table 8).

**Table 5:** Final backward regression models of the relationship between selection criteria and demographics for GEMP students 2005–2012 and academic performance as assessed by overall and yearly WAM for Core units

		***WAM overall (N=219)***	***WAM bridging (N=421)***	***WAM year 3 (N=414)***	***WAM year 4 (N=344)***	***WAM year 5 (N=276)***	***WAM year 6 (N=219)***
***Age (yr)***	Beta	−0.186	−0.147	−0.118	−0.163	−0.266	**−0.136**
	P value	**0.003**	**0.001**	**0.007**	**0.001**	**<0.001**	**0.047**
***Sex (F=0/M=1)***	Beta			−0.078		−0.155	
	P value			0.080		**0.005**	
***GPA at entry***	Beta	0.369	0.347	0.354	0.303	0.159	0.155
	P value	**<0.001**	**<0.001**	**<0.001**	**<0.001**	**0.004**	**0.022**
***Interview score***	Beta	0.159			0.092	0.109	0.168
	P value	**0.007**			0.069	**0.043**	**0.011**
***Health/Allied health background***	Beta	0.272	0.168	0.194	0.278	0.272	0.267
	P value	**<0.001**	**<0.001**	**<0.001**	**<0.001**	**<0.001**	**<0.001**
***Humanities/Law/Business/Commerce background***	Beta	−0.170	−0.127			−0.111	−0.118
	P value	**0.006**	**0.003**			**0.046**	0.081
***Physical sciences background***	Beta						
	P value						
***Rural entry***	Beta	0.104			0.148		0.113
	P value	0.092			**0.005**		0.090
***IRSAD decile (0=1-8, 1=9-10)***	Beta	0.122	0.107	0.134	0.091		
	P value	**0.042**	**0.010**	**0.002**	0.068		
***GAMSAT score***	Beta	0.348	0.286	0.246	0.203	0.283	0.157
	P value	**<0.001**	**<0.001**	**<0.001**	**<0.001**	**<0.001**	**0.019**
***R***^***2***^		***31.0%***	**30.3%**	**25.4%**	**20.1%**	**26.2%**	**14.8%**

(Significant P-values are boldfaced).

**Table 6:** Final backward regression models of the relationship between selection criteria and academic performance in specific ‘knowledge’-based units

		***Pathology, pharmacology and microbiology (Level 3) (N=414)***	***Science and practice of medicine (Level 4) (N=344)***
***Age (yr)***	Beta	−0.084	−0.141
	P value	0.062	**0.005**
***Sex (F=0/M=1)***	Beta		
	P value		
***GPA at entry***	Beta	0.336	0.278
	P value	**<0.001**	**<0.001**
***Interview score***	Beta		
	P value		
***Health/Allied health background***	Beta	0.156	0.240
	P value	**0.001**	**<0.001**
***Humanities/Law/Business/Commerce background***	Beta	−0.079	
	P value	**0.080**	
***Physical sciences background***	Beta		
	P value		
***Rural entry***	Beta		0.183
	P value		**<0.001**
***IRSAD decile (0=1-8, 1=9-10)***	Beta	0.136	
	P value	**0.002**	
***GAMSAT score***	Beta	0.265	0.253
	P value	**<0.001**	**<0.001**
***R***^***2***^		**24.8%**	**18.4%**

(Significant P-values are boldfaced).

**Table 7:** Final backward regression models of the relationship between selection criteria and academic performance in specific ‘clinically-based units

		***Foundations of clinical practice (Level 3) (N=414)***	***Clinical skills (Level 5) (N=276)***	***General practice (Level 5) (N=276)***	***Obstetrics & gynaecology (Level 5) (N=276)***
***Age (yr)***	Beta	−0.138	−0.243	−0.160	−0.294
	P value	**0.002**	**<0.001**	**0.006**	**<0.001**
***Sex (F=0/M=1)***	Beta	−0.166	−0.111	−0.141	−0.146
	P value	**<0.001**	**0.047**	**0.016**	**0.010**
***GPA at entry***	Beta	0.295		0.133	0.197
	P value	**<0.001**		**0.023**	**0.001**
***Interview score***	Beta			0.172	0.120
	P value			**0.003**	**0.032**
***Health/Allied health background***	Beta	0.189	0.214	0.217	0.184
	P value	**<0.001**	**0.001**	**<0.001**	**0.001**
***Humanities/Law/Business/Commerce background***	Beta		−0.118		
	P value		**0.041**		
***Physical sciences background***	Beta				0.108
	P value				0.054
***Rural entry***	Beta				
	P value				
***IRSAD decile (0=1-8, 1=9-10)***	Beta	0.116		0.124	
	P value	**0.010**		**0.029**	
***GAMSAT score***	Beta	0.143	0.313	0.180	
	P value	**0.003**	**<0.001**	**0.002**	
***R***^***2***^		**20.1%**	**20.0%**	**15.9%**	**19.4%**

(Significant P-values are boldfaced).

**Table 8:** Final backward regression models of the relationship between selection criteria and demographics for GEMP students 2005–2012 and academic performance as assessed by overall and yearly WAM for Core units

		***WAM overall (N=219)***	***WAM bridging (N=421)***	***WAM year 3 (N=414)***	***WAM year 4 (N=344)***	***WAM year 5 (N=276)***	***WAM year 6 (N=219)***
***Age (yr)***	Beta	−0.205	−0.149	−0.120	−0.150	−0.269	−0.132
	P value	**0.002**	**0.001**	**0.009**	**0.004**	**<0.001**	0.057
***Sex (F=0/M=1)***	Beta			−0.081		−0.131	
	P value			0.071		**0.018**	
***GPA at entry***	Beta	0.277	0.347	0.354	0.287	0.138	0.139
	P value	**<0.001**	**<0.001**	**<0.001**	**<0.001**	**0.012**	**0.035**
***Interview score***	Beta	0.151					0.142
	P value	0.012					**0.030**
***Health/Allied health background***	Beta	0.264	0.165	0.192	0.278	0.267	0.255
	P value	**<0.001**	**<0.001**	**<0.001**	**<0.001**	**<0.001**	**<0.001**
***Humanities/Law/Business/Commerce background***	Beta	−0.189	−0.122			−0.128	−0.126
	P value	**0.004**	**0.006**			**0.026**	0.068
***Physical sciences background***	Beta						
	P value						
***Rural entry***	Beta	0.108			0.128		
	P value	0.083			**0.014**		
***IRSAD decile (0=1-8, 1=9-10)***	Beta	0.122	0.105	0.132	0.094	0.093	
	P value	**0.042**	**0.011**	**0.002**	0.062	0.080	
***GAMSAT 1: Reasoning in the humanities and social sciences***	Beta	0.140			0.116	0.228	0.133
	P value	**0.045**			**0.034**	**<0.001**	**0.051**
***GAMSAT 2: Written communication***	Beta	0.150	0.154	0.142			
	P value	**0.026**	**0.001**	**0.002**			
***GAMSAT 3: Reasoning in the biological and physical sciences***	Beta	0.218	0.242	0.213	0.116	0.119	
	P value	**0.002**	**<0.001**	**<0.001**	**0.039**	0.056	
***R***^***2***^		**31.5%**	**30.5%**	**26.0%**	**19.1%**	**27.3%**	**13.2%**

(Significant P-values are boldfaced).

## Discussion

The findings of this study indicate that a careful evaluation of the predictive validity of selection factors for graduate entry into medical schools needs to consider as comprehensively as possible the background demographic factors that may simultaneously impinge on both academic performance and the selection factors themselves.

In particular, our observation of poorer performance in GAMSAT Section 1 and 3 but subsequent better academic performance during the course by those from health/allied health disciplines but better performance in GAMSAT Section 1 and 2 and subsequent poorer academic performance during the course by those from a humanities background would serve to underestimate the predictive validity of the score in GAMSAT and its component sections if not taken into account.

Our findings on performance in the GAMSAT by discipline mirror those observed in the 8580 candidates who sat the GAMSAT in 2012 [3]. In that cohort, for overall GAMSAT score those from a physical science background were also the highest performers while those from health sciences and nursing backgrounds were the weakest performers. Similarly, students from arts/social sciences backgrounds were not surprisingly the highest performers in both GAMSAT Sections 1 and 2 while those from a physical science background were the highest performers in Section 3.

It has been observed for more than 3 decades that an undergraduate science background dictates better academic performance in the earlier years of a medical course but that this effect attenuates by the final year of medical school [13]. In the present study there was similarly weaker academic performance by those from a non-science background at levels 2 and 5 with this effect no longer significant by level 6 of the course. In an Australian context those from the health professions have been found to perform better than those with a biomedical science background [14]. In the current study we again observed that those from health-related backgrounds performed better throughout the course, and that this outcome was seen in both knowledge-based and clinically-based units.

In this study we have also been able to consider possible confounding by socio-economic background in relation to the predictive validity of our selection factors. With respect to academic performance there was a negative effect of coming from a more disadvantaged socioeconomic background which attenuated as the course progressed. In Australia and elsewhere, there is an extensive literature indicating weaker academic performance in university in those from disadvantaged backgrounds when socio-economic indicators such as level of wealth, type of high school attended, home location etc. are linked to grades subsequently achieved [15]. These studies have predominantly been undertaken in relation to undergraduate students with fewer insights into whether similar influences carry over into graduate programs of study. Students from independent fee-paying high schools in Australia have higher academic achievement at exit from high school as assessed by Tertiary Entrance Rank than those from government high schools [16]. However subsequent academic performance at a tertiary level is weaker in those from non-government schools, attributed to an enriched teaching and learning environment at high school but a more level playing field once the students are in a university setting [17]. This so-called immersion phenomenon may have been also operative in our graduate cohorts and at least in part offer an explanation for the stronger correlations of the IRSAD decile with academic performance in levels 2 and 3 of the course, with weaker or absent relationships later in the course.

Socio-economic determinants of performance in attribute-based admission criteria for medical schools are now also increasingly recognised. Again at a mainly undergraduate level, we have shown that scores in the UMAT (widely used in Australia and New Zealand for admission to several of the health professions), are lower in students from lower socio-economic deciles, lower in students of non-English speaking background, as well as those from government versus independent schools and those of indigenous background [10]. A recent report on 8,180 individuals who sat the UKCAT in 2009 [9] indicated significant influences of age, ethnicity, school type attended and socioeconomic background on prior academic performance (as assessed by standardised A-level tariffs) but no influence of gender or English as a second language. In contrast all of these variables were significant determinants of UKCAT performance [9]. In a recent Canadian study a measure of socio-economic status linked by postcode to community size and income levels was utilised in applicants to 6 medical schools [7] and an association between lower performance in the MCAT in those from smaller communities was identified but no relationship with income levels was seen. Lower MCAT scores in applicants of self-declared aboriginal origin was also found. Academic performance as measured by GPA was linked to income levels but not to community size. Interview scores were unrelated to either socio-economic measure. In the current study with much smaller numbers we were unable to identify a significant relationship between the IRSAD score and any of our entry selection criteria.

The results from linear regression modelling suggested that each section of GAMSAT variously contributed to the relationship of the total GAMSAT score to academic performance at different stages of the course. The score in GAMSAT 1 (Reasoning in the humanities and social sciences) emerged as a predictor at levels 4 to 6. The score in the GAMSAT Section 2 score (Written communication) although uncorrelated with academic performance in univariate analysis, emerged as a significant predictor in the early levels of the course after controlling for background discipline in linear regression. The Section 3 GAMSAT score (Reasoning in the biological and physical sciences) was the strongest and most consistent GAMSAT section in predicting academic performance with this extending through to Level 5. That this relationship was stronger earlier in the course was unsurprising given the science-oriented nature of the bridging course and a Level 3 curriculum which is pre-clinical, science-oriented and assessed mainly by formal examinations. This section of GAMSAT has been the one most consistently linked to course outcomes in other predictive validity studies [2,4,5].

Prior academic performance as assessed by GPA at entry was the strongest predictor of subsequent academic performance, particularly in the earlier part of the course. This again comes as no surprise given the ‘academic’ nature of the criterion variables in these earlier levels. Such an effect of previous academic achievement is consistent with previous research findings in medical courses [18,19] and specifically in Australian graduate medical programmes [4,5]. The pattern seen here also follows the established trend of diminishing in magnitude in the latter levels of the course.

The amount of variance accounted for in these analyses is slightly higher than those reported by other researchers [4,5]. Furthermore the estimates reported in this study are uncorrected for restriction of range and would have been anticipated to have been even higher had this been done. Based on other studies, it was expected that the accounted variance would be in the range 20 – 25%, however the majority of the values in this study are 25% or higher. This appears to be due to the inclusion of both an indicator of socioeconomic background together with the background discipline variables, particularly the Health/allied health background. In spite of accounting for a number of socio-demographic factors the correlations between our selection scores and academic performance remained relatively weak and similar to levels reported by others [2,5]. Range restriction in the selection scores [20], the specific design and original purpose of each selection instrument and non-cognitive components within the interview score make low correlations with academic performance a likely outcome in predictive validity studies [21], and it has been recently well-argued that the assumption that high-positive correlations are good or even expected in predictive validity studies requires more critical reflection [21].

There were no significant univariate associations of overall academic performance with the interview score. This was not unexpected given that in our larger school-leaver study [11] such effects were seen predominantly in the clinical years (Levels 4 – 6). The observation of an emerging independent relationship to the interview score in the multivariate regression models at levels 4 and 5, and especially in the clinically-based units, is encouraging in terms of the predictive validity of our interview process. However, the relatively lower numbers in the data for Levels 4 – 6 in the current study mean that the relative strength of predictors for these latter clinical years is yet to be fully determined.

### Limitations of the study

The relatively small overall numbers, the different sizes of each annual cohort as well as slight variation over the years in GAMSAT scores, interview scores and GPA at entry for each cohort are limitations in this study. We did not make any corrections for multiple comparisons in the regression models and have made no correction for range restriction. With respect to the former utilising a backwards regression approach minimised the number of predictor variables in the final models. With respect to correction for restriction of range of the criterion variables, this usually results in higher correlations and therefore higher levels of significance in predictive validity studies [20]. The use of an individual’s postcode as a surrogate for socio-economic status imputes an index based on the level of socio-economic disadvantage for all people living in a defined area and may not be truly reflective of socio-economic status for each individual. A proportion of candidates may have been living in student dormitories or lodgings rather than their usual place of residence and this may have weakened the true underlying strength of the associations we have reported. However, aggregating 21 socioeconomic indicators into a single index and then further aggregating by postcode could reduce the variance associated with each indicator and may inflate the strength of the associations reported. Finally socio-economic status linked to an area is not static over time and we have used the 2006 SEIFA codes over a period that spans 2005 to 2012 again potentially weakening the relative accuracy of imputed socio-economic status.

## Conclusion

The outcomes of this study extend the results of Coates [4] and confirm that GPA at entry and the GAMSAT score together predict outcomes not only in the early stages of a graduate-entry medical programme but throughout the course. They highlight the contribution of discipline background and other socio-demographic factors in dictating performance in both the selection factors themselves as well as academic performance during a medical course. The high-stakes nature of the GAMSAT as a major criterion for potential selection of candidates for interview for medical school admission demand that it continue to be appropriately validated as a selection instrument for graduate-entry programmes.

## Competing interests

The authors have undertaken this study in the course of their employment, with no funding from any other source, and have no conflict of interest to declare.

## Authors’ contributions

IP contributed to the conception and design of the study, acquisition, analysis and interpretation of the data; and final revision of the manuscript. AM contributed to the conception and design of the study, interpretation of the data and the initial drafting and final revision of the manuscript for important intellectual content. Both authors read and approved the final manuscript.

## Pre-publication history

The pre-publication history for this paper can be accessed here:

http://www.biomedcentral.com/1472-6920/14/31/prepub
